# Enhancing Postgraduate Learning and Teaching: Postgraduate Summer School in Dairy Science

**DOI:** 10.1155/2014/409549

**Published:** 2014-01-16

**Authors:** Pietro Celi, Gianfranco Gabai, Massimo Morgante, Luigi Gallo

**Affiliations:** ^1^Dairy Science Group, Faculty of Veterinary Science, University of Sydney, PMB 4003, Narellan, NSW 2567, Australia; ^2^Melbourne School of Land and Environment, The University of Melbourne, Parkville, VIC 3010, Australia; ^3^Department of Comparative Biomedicine and Food Science, University of Padova, Viale dell'Università 16, 35020 Legnaro, Italy; ^4^Department of Agronomy Food Natural resources Animal & Environment, University of Padova, Viale dell'Università 16, 35020 Legnaro, Italy; ^5^Department of Animal Medicine Production and Health, University of Padova, Viale dell'Università 16, 35020 Legnaro, Italy

## Abstract

Dairy science is a multidisciplinary area of scientific investigation and Ph.D. students aiming to do research in the field of animal and/or veterinary sciences must be aware of this. Ph.D. students often have vast spectra of research interests, and it is quite challenging to satisfy the expectation of all of them. The aim of this study was to establish an international Ph.D. training program based on research collaboration between the University of Sydney and the University of Padova. The core component of this program was a two-week Postgraduate Summer School in Dairy Science, which was held at the University of Padova, for Ph.D. students of both universities. Therefore, we designed a program that encompassed seminars, workshops, laboratory practical sessions, and farm visits. Participants were surveyed using a written questionnaire. Overall, participants have uniformly praised the Summer School calling it a rewarding and valuable learning experience. The Ph.D. Summer School in Dairy Science provided its participants a positive learning experience, provided them the opportunity to establish an international network, and facilitated the development of transferable skills.

## 1. Introduction

Internationalization of higher education refers to institutional arrangements set up by governments, universities, and education agents that involve the delivery of higher education services in two or more countries. For teaching and research, effective and appropriate national and international networks are essential. In some cases, the academic enterprise will depend on an international network of collaboration, while, in other cases, it is difficult to imagine how anything much could be achieved without such a network. Academics often establish their own networks, and the best role of the institution may often be facilitation, following after good leads, and reducing bureaucratic drag and friction. Individual research collaborations between academics from different countries are important; however, formal collaborations between universities that involve curriculum and program development are growing and likely to expand in the years ahead. Such arrangements could include short-term placements in overseas laboratories, arrangements for registration for specialized Masters level courses, teaching fellowships to widen experience, and joint supervision and coqualification arrangements, such as the increasingly popular cotutelle programmes. If appropriately organized, such bilateral arrangements can foster research collaboration, as research communities build interests and respect through the shared supervision and training of one another's postgraduate students.

Accompanying the increased international mobility of students and academic staff, universities have taken steps in recent decades to enhance the international content of their programs and they have also established dedicated portfolios like the International Program Development Fund (IPDF) which provides funding to support initiatives in internationalization. The IPDF program supports initiatives for innovative and sustainable programs built around collaborative research and/or learning and teaching initiatives linking the University of Sydney with the world's leading academic institutions.

### 1.1. Veterinary Scientists

The veterinarian scientist is an endangered species [1]. Very few veterinarians opt for a research focus and even less focus on agricultural research. Tertiary institutions, thus, need to attract new veterinary students to consider a research career. One approach would be to provide money and mentors (“the two Ms”) [1]. Money is a critical component in providing both incentives for training and suitable posttraining opportunities. With increased tuition rates and cost of living, access to scholarships has a great effect on accessibility and on who attends the university. In the United States, some institutions and many states have recognized that accessibility is an issue and are taking specific actions such as waiving tuition for low-income students; others have established scholarship programs funded with state lottery income as a means to offset some of the effects of tuition increases [2]. Mentors are role models and are equally important. It is not our intent to discuss the role of “the two Ms” in successfully attracting and retaining veterinarian scientist in the agricultural research community; however, we believe that by establishing an international scientific network between Ph.D. students and academic staff might “rescue the veterinary scientist from extinction” [1]. Internationalization of the universities and their postgraduate programs is a complex task and it is a process well beyond the current program presented here. It requires the establishment of a network of universities open to collaborate in strategic efforts aimed at preparing students to compete and collaborate in today's global society.

Given the intimate connection between research and postgraduate education, we have built reciprocal relationships between the University of Sydney and University of Padova to address our research needs in Veterinary Science (Dairy Science). We have also established international collaborations to enhance postgraduate training and development by organizing a Summer School in Dairy Science for Ph.D. students.

### 1.2. Dairy Science

Dairy science is a multidisciplinary area of scientific investigation. Ph.D. students aiming to do research in the field of animal and/or veterinary sciences need to be aware of this. When we glance over the index of the Journal of Dairy Science, the top-ranked journal in the Dairy and Animal Science category, it is easy to realize that articles are grouped into the following categories: Dairy Foods, Physiology and Management, Nutrition, Feeding and Calves, Genetics and Breeding, and Our Industry Today. In other words, dairy science is a complex but integrated area of scientific investigation, which Ph.D. students aiming to do research in the field of animal and/or veterinary sciences cannot ignore. Ph.D. students often have vast spectra of research interests, and it is quite challenging to satisfy the expectation of all of them. The professional expectations of the dairy industry require advanced skills from those who will work in it both now and in the foreseeable future [3]. Also, it is important that veterinarian and animal scientist acquire the required competencies to work across several disciplines in order to offer the dairy industry leadership and guidance in meeting its goals [4].

It is clear that, for learning organizations, globalization is both a powerful challenge and an opportunity [5]. Today, almost every country has three ambitions regarding higher education [6]: to admit more students to a university, to improve higher education to compete in an international market, and to offer university education to students disadvantaged because of their social, cultural, or ethnic background. Veterinary colleges are beginning to recognize the tremendous need for educating students in international matters [7, 8]. The purpose of internationalizing the postgraduate curriculum in veterinary science within our academic institutions was twofold. Our intent was to convey an understanding of other veterinary systems, including different cultures and economic systems to our postgraduates having in mind that globalization will, sooner or later, impact everyone in the veterinary profession. This internationalization process and the awareness of globalization require cultural changes within the tertiary institutions, so that postgraduate students can interact in an environment where their interest in local, national, and international issues is stimulated. The second purpose was that there will be increasing international opportunities in some fields of veterinary medicine, and, specifically, public and animal health, ecology, food quality and safety, and animal production (nutrition and reproduction), and, consequently, there is a need to provide appropriate training to allow graduates to function in these areas. In our opinion, these two purposes seem to complement each other. As students are encouraged and facilitated in career paths and specific training involving international experiences is provided, the culture within universities will shift towards a global outlook, and, as the environment is more encouraging for international subjects, increasing numbers of students migrate toward international career interests [7].

Thus, the aim of this paper is to describe the program goals, strengths and weaknesses of the Ph.D. Summer School in Dairy Science jointly run by the University of Sydney and the University of Padova, and to discuss the implications of the Program to the internalization of postgraduate veterinary education in general and to the field of dairy science in particular.

## 2. Materials and Methods

### 2.1. The Ph.D. Summer School in Dairy Science

The aim of the Ph.D. Summer School project was to establish an international program based on research collaboration between the University of Sydney and the University of Padova. The core component of this program was a two-week residential, which was held at the University of Padova, for Ph.D. students of both universities. The Postgraduate Summer School in Dairy Science was presented as an opportunity for a concentrated and advanced learning experience for Ph.D. students enrolled in the Faculty of Veterinary Science (University of Sydney) in the Ph.D. School of Animal Science (University of Padova) and the Ph.D. School in Veterinary Science (University of Padova).

The participants were exposed to leaders in the field of dairy science. Additionally, they had the opportunity to interact with each other. This was achieved not only during the several activities of the Summer School but also during the coffee and meals breaks. Evening meals were also organized on 3-4 occasions to provide additional socialization and interaction opportunities. We intended to provide Ph.D. students and top dairy scientists opportunities to create friendships and networks of colleagues to the benefit of the profession and the industry. Moreover, by inviting renowned lecturers from all over the world, the educational program is held in an international atmosphere enhancing exchange of scientific ideas and technological advances within the fields of dairy science.

Therefore, we designed a program that encompassed seminars, workshops, laboratory practical sessions, and farm visits. The modules were presented by experts in the field and covered the following topics: dairy production systems in Italy, Argentina, and Australia; stem cells in animal science and veterinary medicine; milk quality; alternative use of milk and milk by-products; fertility and sources of reproductive wastage in dairy cows; health management of the dairy herd; new approach to the evaluation of feed and diets of dairy cows; and ecology of farming systems (Table 1). The modules were taught entirely in English which is considered the international language of the scientific community. Prior to each module, lectures provided a reading list which was forwarded to the participants for their preparation.

The activities have been designed so that the Ph.D. students would meet the following learning objectives: (1) outline the differences in dairy production systems in Italy, Australia, and around the world; identify how the various husbandry and management techniques can impact on dairy cows' welfare, health, and production and recommend procedures to minimize these impacts; (2) identify factors affecting milk characteristics and explore alternative industrial possibilities to exploit milk by-products; (3) communicate and interact competently with fellow Ph.D. students, scientists, and producers; and (4) develop a scholarly and integrative approach to research in animal and veterinary science and widen the perspective of the Ph.D. students about biology applied to animal and veterinary sciences. Participation to the modules was voluntary with an average of 25 students attending each of the modules.

The integrated approached that we designed does not mean that all staff involved had equivalent roles, responsibilities, and competencies. It means that the core responsibilities of each staff (and Ph.D. student) involved in the Summer Schools should include some level of engagement and responsibility in each module of the Summer School. We believed that the pursuit of an integrated approach in Dairy Science will genuinely engage Ph.D. students in the learning and teaching activities designed in the Summer School and ultimately will provide them with a positive learning experience.

### 2.2. Evaluation

Participants were asked to do an evaluation at the end of the Summer School by means of a dedicated survey. The survey consisted of 32 questions (Table 2); participants were asked to indicate the extent to which they agreed or disagreed with the statements reported in each question, using a five-point Likert-type scale, where 1 = strongly disagree (SD), 2 = disagree (D), 3 = neutral (N), 4 = agree (A), and 5 = strongly agree (SA). Participants also had the opportunity to provide feedback by writing a comment explaining their rating for each item and providing suggestions for improvement. The survey was distributed at the end of the last lecture scheduled on the last day of the Summer School, giving students approximately 45 minutes to complete it. The response rate was 64% (32/50). For this sample size, the observed response rate is consistent with published guidelines [9]. Results were compiled and cumulative percentages for SD + D, N, and A + SA responses and average scores have been reported in Table 2.

## 3. Results and Discussion

The feedback gathered, together with informal comments, provided important ideas that could be used to improve future programs. Overall, participants have praised the Ph.D. Summer School calling it a rewarding and valuable learning experience (Table 2). Some of the expressions used in support of their evaluation include “the satisfaction level is high; I think that the overall work done was of a high quality, both professors and Ph.D.,” “outstanding people, presentations and opportunities,” “great group of people, good science, positive atmosphere,” “good program, possibility to see researchers of other countries how perform their research, possibility to speak with foreign Ph.D. students, to see the different aspects from ours,” “contacts, the name of the game, this was on outstanding scaffold to become involved in the broader research culture.” Indeed, the results gathered by the survey indicates that the majority of participants found the Ph.D. Summer School as a useful part of their learning experience (67%), that the Ph.D. Summer School was intellectually stimulating (60%), that “the Ph.D. Summer School has sharpened their analytical skills” (53%), that “the Ph.D. Summer School has stimulated their enthusiasm for further learning” (60%), that “the Ph.D. Summer School program was good” (67%), and that, overall, were satisfied with the quality of this course (53%).

It is important to note that some questions were ranked relatively low (2.69–3.08) for a Likert-scale assessment, namely, “the Ph.D. Summer School has helped me develop my ability to work as a team member,” “the Ph.D. Summer School has helped me develop my problem-solving skills,” “the Ph.D. Summer School has helped me develop my skills in written communication,” “the Ph.D. Summer School fostered my personal and intellectual independence,” and “As a result of this course, I feel confident about tackling unfamiliar problems.” It seems that these scores might have been the consequence of not all students taking part in modules that encompassed group or written activities. This is an important observation as it will help us to better align the different modules with the intended learning outcomes developed for the Ph.D. Summer School. For the other questions, two students in particular indicated that “the fields of the Summer School were too much different from mine” or that “because my field of research is another one, it is really different” or “I'm sorry, the course was very well organized, but it is not useful obligate all Ph.D. students of our school to participate. I wasted time that I could spend to work on my project.” These comments highlight the fact that these two students did not engage with the learning and teaching activities offered during the Ph.D. Summer School as a result of being strongly encouraged to attend this program despite the fact that participation was on a voluntary basis. It is obvious, thus, that designing these programs poses some challenges for the organisers. We propose that future activities would also have to include a “train the trainer” session where all the facilitators taking part in the program and Ph.D. supervisors can receive adequate training to then be able to adequately perform their roles that are to create a safe and stimulating learning environments, help participants to gain academic and personal insight from learning activities, and to facilitate the development of transferrable skills and lifelong learning.

Of note is the comment made by all the invited international speakers of how much they enjoyed the program and being with a group of people that shared their same passion and interest for science and the dairy industry. All speakers expressed strong interest in participating in future activities and most of all they clearly indicated that they would like their Ph.D. students to participate in the next Summer School.

Many of the key strategies adopted in the Ph.D. Summer School in Dairy Science seem to be in alignment with those outlined by Taylor [10]. We have provided an enhanced social networking for international students including links with international and national staff. By incorporating community and social interaction into the learning environment, we can enhance learner motivation, activity, and self-esteem. Enhanced networking is facilitated in the broadest arenas of academic disciplines through involvement with diverse new contacts, academic staff, and peers. Such engagement assists in appreciating diversity and the development of global perspectives, thus serving as a model for dissemination of good practice more widely across academic provision. As a consequence, student and staff horizons are broadened within the curriculum [11]. Students were able to participate in a number of learning modules within a program that was carefully planned by the Ph.D. Schools in Veterinary and Animal Science (University of Padova) and by the University of Sydney. Particular emphasis was placed on the quality of the learning experience provided and on maintaining and improving the academic standard of the Summer School. We believe that the Ph.D. Summer School in Dairy Science is an important emerging strategy for internationalization of learning and teaching in tertiary institution.

### 3.1. Outcomes and Implications

The outcomes of the Ph.D. Summer School in Dairy Science are diverse and far-reaching, and, although they are particularly relevant to Australian and European Universities, they could be considered as an example by other institution wishing to implement a similar program for Ph.D. students. An important outcome is the formation of an international network of dairy scientists and Ph.D. students. Close contact between participants and scientists is developing. Two Ph.D. students from the University of Padova have attended the annual Dairy Research Foundation Symposium (September 16-17, 2009). The two students presented a paper during the Young Scientist sessions and they also had the possibility to visit the state-of-the art labs and dairy farm that we have in place at the University of Sydney. The exchange of Ph.D. students between the University of Sydney and the University of Padova is continuing and we are currently exploring the possibility of setting-up a Ph.D. in cotutelle programme. This is probably one of the most important outcomes as it will lead to networking between Ph.D. students of different tertiary institutions but will also create a collaborative network between research institutions and academic staff involved in dairy science research. It has to be noted though that a controversial aspect of moving toward an international model of postgraduate veterinary medical education is that it could lead to calls for the award and recognition of joint degrees or awards. Joint degrees resulting from cooperation among several higher education institutions located in different countries, although appearing threatening to some institutions, have considerable potential [12]. The European Union has already moved in this direction with the Erasmus World program (Socrates Erasmus Programme) [13] including the development of about 90 interuniversity networks to provide 250 joint master's courses to students around the world. Considering that one of the indices of maturity and leadership of a university department or program is its international linkages [14], programs like the Ph.D. Summer School provide an excellent opportunity for tertiary institutions to raise their international profile.

Another outcome of this Summer School in Dairy Science is that it opens up a door in the European Union (EU) for Australian tertiary institutions. The EU offers a wide range of research grant opportunities that mainly promote research mobility for early career researchers and postgraduate students. As international activities increase in their popularity and importance, they will increasingly affect the core educational program of veterinary schools. These institutions will have to address the international dimension of their curricula in ways that reflect their values, priorities, opportunities, and revenues [15]. We believe that educating students in such a way that their perspectives are limited to veterinary practice in the context of their own country is no longer sustainable. When we design and implement new learning and teaching activities, we must ask ourselves if we are doing enough to prepare our students to succeed in a globalized world. Are the students learning the skills necessary to be leaders of the veterinary profession within the 21st-century global village in which we live? [8]. We should keep in mind that international education in veterinary science goes well beyond increased spatial mobility or transfer of curricula from one school to another. The ability to benefit from international programs while maintaining a national character is a key challenge for higher education institutions for the future [16]. The concept of globalization of veterinary medicine education has emerged from the accumulating evidence of changing needs and expectations and that current programs that might provide veterinary leadership on these issues are needed [17]. Global veterinary leadership is a concept that addresses the critical need for veterinarians with knowledge not only in areas traditionally associated with veterinary medicine but also in the global issues of food safety, trade, and information management [17]. We must expand opportunities for veterinary scientists to develop an understanding of global interdependence and the commonality of veterinary and food safety and security problems as well as the skills to help solve them. Programs like the Ph.D. Summer School can provide a framework for harmonizing veterinary and animal sciences education. Also, experiencing the cultural aspects of veterinary and animal science in another country, as influenced by education, politics, and economics, contributes directly to the student's discovery of critical, global understanding [17].

Finally, Ph.D. programs are often characterized by a high degree of specialization where students mostly focus on their research project while a vertical integration within the program (Dairy Science in this case) and on transferrable skills is often lacking or there is very little emphasis on it. Transferable programmes for Ph.D. students are becoming more popular within the European Union and North America [18]. The TRANS-DOC programme aims to provide a leading example of an innovative way of providing Ph.D. students with the knowledge and tools to enhance their employability and develop the skills needed to respond to a society in constant change. The TRANS-DOC programme has been developed to ensure that Ph.D. students are informed about generic skills such as collaboration skills (working effectively with others and working multiculturally and cross-disciplinarily), communication skills (communicating your research to nonexperts and the media, presentation skills, body language, and rhetoric), knowledge transfer and entrepreneurship (project management, fund raising, creativity, and generation of ideas), intrapersonal skills (self-awareness, time management, stress management, and work/life balance), networking, professionally and personally, and ethics and social responsibility [18]. We believe that the Ph.D. Summer School in Dairy Science has provided its participants the opportunity to develop such skills; however, it would be ideal if more opportunities would be provided to Ph.D. students during the course of their candidature.

## 4. Conclusions

The organizers of the Ph.D. Summer School in Dairy Science together with the lecturers involved in the program are the driving forces of this endeavor and we are comforted by the success of our young Ph.D. students. The key to the contribution that international academic networks make to regional and local challenges in social responsiveness is the realization of the value of comparative and multidisciplinary studies. Our aspiration was to help prepare a new generation of veterinarians and animal scientists with a more global outlook and a better appreciation of the role of veterinary medicine and animal science in dairy science. By offering the Ph.D. Summer School in Dairy Science, we intended to empower postgraduate students with a global perspective of the issues that veterinarians and animal scientists have to deal with on a daily basis. In our view, this is the way to provide Ph.D. students with a holistic approach to science in general and to dairy science in particular. Our long-term objective is advance international cooperation between research institutions as this would result in a better use of resources, in a greater research output, and would offer a better learning experience to postgraduate students. Ultimately, we would hope that the increased internationalization of postgraduate programs like the Ph.D. Summer School in Dairy Science would promote the submission of international grant applications, facilitate the recruitment of new staff, and provide the dairy industry with true international leaders. Therefore, the outcomes of this program are valuable for veterinary and animal scientists, employers of veterinary and animal scientists, current and prospective Ph.D. students, tertiary education providers, and funding agencies and have the potential to help direct future efforts in veterinary postgraduate education.

## Figures and Tables

**Table 1:** Modules offered during the Ph.D. summer school in dairy science.

Module	Lecturer(s)
Dairy production systems	Dr. Pietro Celi, Faculty of Veterinary Science, University of Sydney Santiago Farina (Ph.D. student), Faculty of Veterinary Science, The University of Sydney Professor Luigi Gallo, Department of Animal Science, Faculty of Veterinary Medicine, University of Padova

Stem cell research in dairy science	Dr. Marco Patruno, Department of Veterinary Experimental Sciences, University of Padova Professor Anthony Capuco, Bovine Functional Genomics Lab, Beltsville, MD, USA

Milk quality and dairy technology	Professor Luigi Gallo, Department of Animal Science, University of Padova Professor Martino Cassandro, Department of Animal Science, University of Padova

Bioactive peptides in milk	Professor Enrico Novelli, Department of Public health, Comparative Pathology and Veterinary Hygiene, University of Padova Dr. Didier Dupont, National Institute for Agricultural Research (INRA), Rennes, France

Reproduction management in the modern dairy cow	Dr. Pietro Celi, Faculty of Veterinary Science, University of Sydney Shelley Underwood (PhD student), Faculty of Veterinary Science, University of Sydney

Health management of dairy herds	Professor Massimo Morgante, Department of Veterinary Clinical Science, University of Padova Professor Michael Doherty, School of Agriculture, Food Science & Veterinary Medicine, Dublin, Ireland

Nutrition management of high producing dairy cows	Professor Lucia Bailoni, Department of Animal Science, University of Padova Dr. Franco Tagliapietra, Department of Animal Science, University of Padova Associate Professor Paolo Berzaghi, Department of Animal Science, University of Padova Associate Professor Luca Rapetti, Department of Animal Science, University of Milano

Ecology of farming systems	Dr. Enrico Sturaro, Department of Animal Science, University of Padova Associate Professor Marco De Liguoro, Department of Public Health, Comparative Pathology and Veterinary Hygiene, University of Padova Professor Karl Fent, University of Applied Sciences, Muttenz, Swiss Federal Institute of Technology (ETH), Zürich, Switzerland

**Table 2:** Question delivered in the Ph.D. summer school evaluation form, cumulative percentages for SD + D (strongly disagree + disagree), N (neutral), and A + SA (agree + strongly agree) responses, and average scores.

Survey questions	SD + D (%)	N (%)	A + SA (%)	Average score
I found the PhD Summer School a useful part of my learning experience	13	20	67	4.00
I found the PhD Summer School intellectually stimulating	0	40	60	3.85
The Ph.D. Summer School has helped me develop my ability to work as a team member	40	33	27	3.08
The Ph.D. Summer School has sharpened my analytical skills	13	33	53	3.85
The Ph.D. Summer School has stimulated my enthusiasm for further learning	13	27	60	3.92
The Ph.D. Summer School has helped me develop my problem-solving skills	53	20	27	2.85
The material provided encouraged me to learn more about the topics covered	40	27	33	3.23
I have learned to explore ideas confidently with other people	20	33	47	3.54
I felt that I benefit from being in contact with active researchers	7	20	73	4.00
I felt part of a group of student and staff committed to learning	20	47	33	3.54
The teaching staff worked hard to make their subject interesting	7	27	67	4.00
The Ph.D. Summer School has helped me develop my skills in written communication	67	7	27	2.69
The Ph.D. Summer School has helped me develop my capacity for research and enquiry	20	53	27	3.25
The PhD Summer School fostered my personal and intellectual independence	33	53	13	3.08
The PhD Summer School has helped me develop my ability to use information effectively	27	27	47	3.62
I was able to explore academic interest with academic staff and fellow students	13	33	53	3.75
The teaching staff of this course motivated me to do my best work	27	7	67	3.92
As a result of this course, I feel confident about tackling unfamiliar problems	27	53	20	3.08
The teaching staff provided additional information relevant to my topic	40	13	47	3.31
The PhD Summer School provided opportunities for me to become involved in the broader research culture	27	33	40	3.33
The PhD Summer School program was good	7	27	67	4.17
Interaction with other postgraduate students was actively encouraged during the Summer School	33	13	53	3.46
The facilities (e.g., classrooms, lecture theatres, studios, labs, and workshops) were adequate for this course	13	13	73	3.92
The learning outcomes of the PhD Summer School were clear to me	7	33	60	3.85
The teaching in this course helped me to learn effectively	40	27	33	3.08
I was motivated to engage with the learning activities in this course	27	40	33	3.31
I can see the relevance of this course to my professional development	40	20	40	3.23
I was stimulated to participate as an active learner in the course	33	33	33	3.31
I have learnt to develop ideas and present them in my written work	47	20	33	3.15
Overall, I was satisfied with the quality of this course	27	20	53	3.62
What were the best aspects of the PhD Summer School? Please explain why these aspects are good				n/a
What aspects are most in need of improvement? Please explain why				n/a
